# MicroRNA162 regulates stomatal conductance in response to low night temperature stress *via* abscisic acid signaling pathway in tomato

**DOI:** 10.3389/fpls.2023.1045112

**Published:** 2023-03-02

**Authors:** Yangyang Li, Yang Liu, Zhenhua Gao, Feng Wang, Tao Xu, Mingfang Qi, Yufeng Liu, Tianlai Li

**Affiliations:** ^1^ Department of Horticulture, Shenyang Agricultural University, Shenyang, China; ^2^ Key Laboratory of Protected Horticulture of Education Ministry and Liaoning Province, Shenyang, China; ^3^ Collaborative Innovation Center of Protected Vegetable Surrounds Bohai Gulf Region, Shenyang, China; ^4^ Tongliao Agricultural Technology Extension Center, Tongliao, China

**Keywords:** microRNAs, ABA, stomata, resistance, cold stress, tomato

## Abstract

MicroRNAs (miRNAs) mediate the degradation of target mRNA and inhibit mRNA translation to regulate gene expression at the transcriptional and post-transcriptional levels in response to environmental stress in plants. We characterized the post-transcriptional mechanism by deep sequencing small RNA (sRNA) to examine how miRNAs were involved in low night temperature (LNT) stress in tomato and whether the molecular mechanism depended on the abscisic acid (ABA) signaling pathway. We annotated conserved miRNAs and novel miRNAs with four sRNA libraries composed of wild-type (WT) tomato plants and ABA-deficient mutant (*sit*) plants under normal growth and LNT stress conditions. Reverse genetics analysis suggested that miR162 participated in LNT resistance and the ABA-dependent signaling pathway in tomato. miR162-overexpressing (pRI-miR162) and miR162-silenced (pRNAi-miR162) transgenic tomato plants were generated to evaluate miR162 functions in response to LNT stress. miR162 deficiency exhibited high photosynthetic capacity and regulated stomatal opening, suggesting negative regulation of miR162 in the ABA-dependent signaling pathway in response to LNT stress. As feedback regulation, miR162 positively regulated ABA to maintain homeostasis of tomato under diverse abiotic stresses. The mRNA of *DICER-LIKE1* (*DCL1*) was targeted by miR162, and miR162 inhibited DCL1 cleavage in LNT response, including the regulation of miRNA160/164/171a and their targets. The DCL1-deficient mutants (*dcl1*) with CRISPR/Cas9 prevented stomatal opening to influence photosynthesis in the ABA signaling pathway under LNT stress. Finally, we established the regulatory mechanism of ABA-miR162-DCL1, which systematically mediated cold tolerance in tomato. This study suggests that post-transcriptional modulators acted as systemic signal responders *via* the stress hormone signaling pathway, and the model at the post-transcriptional level presents a new direction for research in plant abiotic stress resistance.

## Introduction

MicroRNAs (miRNAs) are endogenous non-coding small RNAs (sRNAs) that participate in plant development, organ formation, signal transduction, and stress response (Cai et al., 2009). Although miRNAs do not directly encode proteins, their target genes are activated to promote functions (Jones-Rhoades et al., 2006). miRNAs modulate target genes at the transcriptional and post-transcriptional stages, like Dicer-like (DCL) cleavage functional processes. DCL plays an important role in RNA degradation and regulation of gene expression as a conserved double-stranded RNA specific endonuclease (Ketting et al., 2001). DCL1 mainly cleaves the miRNA precursor to form mature miRNA and participates in miRNA biosynthesis (Gasciolli et al., 2005; Margis et al., 2006; Kravchik et al., 2014b). A previous study showed that miR162 affects post-transcription by regulating DCL1, which is a target gene of miR162 in tomato (Kravchik et al., 2014b). Further, DCL1 is the major producer of miRNAs (Park et al., 2002). In *DCL1*-deficient mutants of tomato, the expression of three miRNAs (miR160, miR164, and miR171a) are decreased, and the expression of their targets are increased (Kravchik et al., 2014a), indicating the function of DCL1 cleavage in miRNA regulation. However, some researchers have developed a DCL1-independent model in miRNA generation and have revealed that the reduction in miR172 causes flowering delay in *dcl1-7* mutant of *Arabidopsis thaliana* (Tsuzuki et al., 2014). Whether the feedback regulation consisted of miRNAs and DCL1 cleavage is still unknown.

miRNAs are considered vital molecular tools for studying stress tolerance of plants (Noman et al., 2017), including low temperature (Chen et al., 2015a), drought (Zhou et al., 2016), and other abiotic stresses (Hackenberg et al., 2015). However, it is still limited and sometimes contradictory about the identity of miRNAs in tomato (Arazi and Khedia, 2022). There are three functional mechanisms of miRNAs in the responsive regulation of plants. The first function is targeting mRNAs by specific cleavage. For example, miR166 cleaves mRNA by the RNA-induced silencing complex (RISC) with the transcription factor (TF) basic region leucine zipper (bZIP) (Castanotto et al., 2009). The second function is inhibiting translation. miR172 is involved in flower development by repressing AP2 protein translation in Arabidopsis (Chen, 2004; Ricci et al., 2011). The third function of miRNA regulation is silencing of related genes. It is indicated that the chromosome structure was changed as genes silenced by miRNA regulated DNA methylation (Ricci et al., 2011). In recent years, more studies have been devoted to elucidating how miRNAs are involved in plant stress response. Under salt stress, miR162a biosynthesis is regulated by pri-miRNA polyadenylation in Arabidopsis (Jones, 2002). Further, miRNAs are involved in post-transcription to affect plant resistance (Sun et al., 2012) and regulate stomatal movement to mediate stress responses; for example, miR169c targets stochastic location model with risk pooling 1 (SlMRP1) to activate stomatal aperture in tomato response to drought stress (Zhang et al., 2011), and miR824 targets AGL16 to affect the development and movement of guard cells in Arabidopsis (Kutter et al., 2007). The finding suggests that sly-miR164a is required for normal fruit development and has negative regulations in tomato cold tolerance by promoting ethylene production (Dong et al., 2022). In addition to abiotic stress, the resistance of *Fusarium oxysporum* f. sp. lycopersici (race 2) has been enhanced in sly-miR482e-knocked down tomato plants (Gao et al., 2021). In protected cultivation during winter, crops such as tomato often experience low temperature stress that limits plant growth and development at night (Liu et al., 2012). However, studies of the association between low night temperature (LNT) stress and miRNA-modulated responses are limited.

Abscisic acid (ABA) is a phytohormone that efficiently responds to multiple abiotic stresses. The ABA-dependent signaling pathway is a common defense mechanism in plants (Roychoudhury et al., 2013). It is indicated that miRNAs such as miR167 and miR413 are regulated by ABA in previous studies (Liu et al., 2009). Meanwhile, miRNAs activated the ABA signaling pathway temporarily in osmotic stress (Jung and Kang, 2007). Both ABA content and sensitivity to salt stress are enhanced in miRNA mutants, and the expression levels of ABA response factors are increased with miRNA deficiency (Zhang et al., 2011). The miR166 targets participated in the ABA pathway as bZIP TFs (Baloglu et al., 2014). Auxin response factor 10 (ARF10) is sensitive to ABA, which was targeted by miR160 in tomato (Liu et al., 2007). Although miRNA plays regulatory roles in the ABA pathway, whether the regulation of defense miRNAs depends on ABA remains unclear. The presence of a feedback relationship between miRNAs and ABA in response to cold stress still needs further study in tomato. A promoter scan of DCL detected numerous *cis*-elements implicated in hormone responses, but it is not clear whether ABA-related TFs affect DCL and miRNA formation in stress resistance. Sucrose non-fermenting 1-related protein kinases 2 (SnRK2) was sensitive to ABA and affected miRNA production by acting on the DCL processing complex in Arabidopsis (Boudsocq et al., 2004; Yan et al., 2017). However, it is unknown whether ABA participates in DCL1 cleavageand directly affects the production of downstream miRNAs in plant response to stresses. The relationship between phytohormones and post-transcriptional regulatory mechanism of miRNAs has become the next research direction in plant stress resistance.

In the present study, we constructed sRNA libraries with four tomato groups, including wild-type (WT) tomato plants and ABA-deficient mutant (*sit*) plants under normal growth conditions and LNT stress. LNT-responsive miRNAs were identified by the ABA signaling pathway in tomato. Based on these results, miR162-overexpressing/-silenced transgenic lines (pRI-miR162/pRNAi-miR162) and DCL1-deficient mutants (*dcl1*) with CRISPR/Cas9 were generated to elucidate how the post-transcriptional process participates in cold tolerance in tomato. Furthermore, we provide a theoretical basis for artificial regulation in tomato cultivation under cold stress and provide ideas for research on miRNAs regulating targets to improve cold resistance in plants.

## Materials and methods

### Plant materials and growth conditions

Seeds of the ABA-deficient mutant sitiens (*sit*) and WT cultivar Rheinlands Ruhm (RR) of tomato (*Solanum lycopersicum* L.) plants were obtained from the Tomato Genetics Resource Center (TGRC; http://tgrc.ucdavis.edu). The *sit* mutant reduces a substantial proportion of ABA-aldehyde to alcohol and is deficient in functional enzymatic activity at the final step of ABA biosynthesis (Harrison et al., 2011). All experiments were completed in the artificial climate chamber. Seeds were germinated in plug trays with natural light and 60% humidity under normal temperature conditions (25°C, 12 h for day/15°C, 12 h for night). Seedlings with the fourth functional leaf fully expanded were transferred to 18 × 18 cm plots to use for experiments. Four sample groups with sixteen tomato seedlings per group were created, including WT tomato plants under normal conditions (WC), WT tomato plants under LNT stress condition (WL), *sit* tomato mutant under normal conditions (SC) and *sit* tomato mutant under LNT stress condition (SL). The LNT treatment condition was set to 12 h/12 h (day/night), which daytime is during 7:00-19:00 with 600 µmol m^-2^ s^-1^ light intensity, the temperature is 25°C, and the nighttime is during 19:00-7:00, the temperature is 6°C for 9 days. The treatment of control was set to 12 h/12 h and 25°C/15°C(day/night), other conditions are the same as LNT.Four biological replicates were used, according to four leaves in the same position of different tomato seedlings in each treatment.

### sRNA isolation and identification

Tomato leaves of each group were collected from 0 to 9 treatment days.Total RNA was isolated from tomato leaves according to the manufacturer’s instructions of the miRNA isolation Kit (TIANGEN, Beijing, China). Concentration of total RNA were determined by OD value. Purity of RNA was obtained by gel electrophoresis (2 μg).

### Deep sequencing of sRNAs

An individual sRNA library was constructed for each sample group. 3’NEXTflex Adenylated Adapter 1 μL was added into 2 μg total RNA and placed in a PCR apparatus at 70°C for 2 min after centrifugation and incubated at 22°C for 2 h. Then, 1 μL primer was added for the reaction: 70°C, 5 min; 37°C, 30 min; 25°C, 15 min. Then, 4 μL 5’NEXTflex Adapter was added, and heated for 2 min at 70°C, and incubated at 20°C for 1 h. After reverse transcription of RNA and PCR amplification of cDNA, all samples were stored at −20°C. Reverse-transcription and PCR amplification was reacted with products after dephosphorylation and ligation with an adapter sequence (Xie et al., 2014). Sequencing was performed on the Illumina HiSeq^TM^ 2000 system at Biomarker Technologies (Beijing, China).

To obtain the clean reads, vector sequences and contaminants were removed from sequences. sRNA distribution and expression were analyzed by Bowtie. Both sRNA tags matching exons and introns of mRNAs or ncRNAs, including rRNA, tRNA, snRNA, snoRNA, and other ncRNAs were deposited into Rfam (Li et al., 2013). Filtered reads were compared with tomato miRNA sequences available from miRBase by a BLAST search (Kozomara and Griffiths-Jones, 2014). Mireap pipeline was used to identify precursor sequences of conserved and novel miRNAs (http://sourceforge.net/projects/mireap). Potential miRNA-target genes were annotated using ITAG2.3. MiRNAs obtained from different samples were compared pairwise to identity differences in expression using a log_2_-ratio scatter plot. Pathway classification was performed on the BMK cloud analysis platform.

### Quantitative RT-PCR (qRT-PCR) analysis

qRT-PCR analysis was performed to determine the expression levels of several randomly selected conserved or novel miRNAs. Primers were coupled in 6 to 8-nts that complement corresponding miRNA at the 3’ end of stem-loop structure, which were designed based on miRNA sequences (**Table S4**). U6 snRNA (X52315) of maize was used as an internal control. SYBR Premix Ex Taq^TM^ Kit was used for qRT-PCR. The 7500 RT-PCR System was performed with a reaction volume of 20 μL, containing 2.5 μL cDNA, 2.5 μL forward/reverse primers, 5 μL of ddH_2_O, and 12.5 μL of 2 × SYBR Green PCR master mix (Applied Bio systems, CA, USA). The reaction mainly included three steps: pre-denaturation (95°C; 10 min), denaturation (95°C; 15 s) followed by 40 cycles, and annealing (60°C; 30 s). The relative miRNA quantities were calculated by Ct (2^−ΔΔct^) method (Livak and Schmittgen, 2001).

### Generation of miR162 and DCL1 transgenic tomato plants

**Figure 1:**
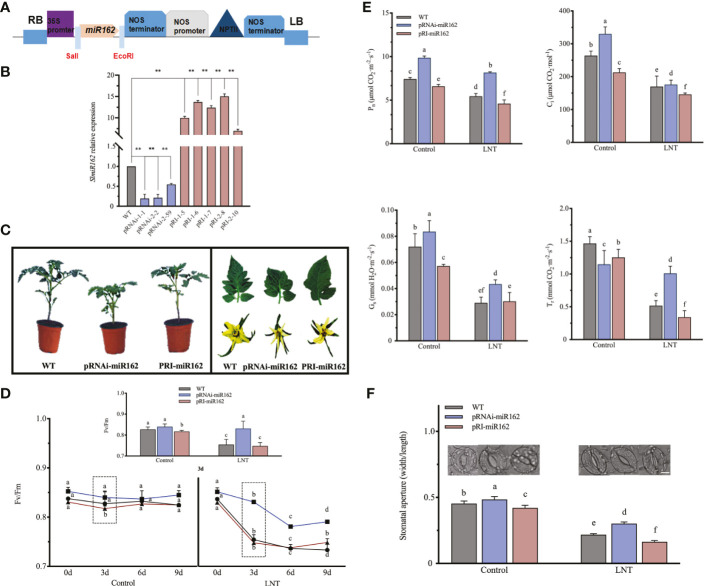
Photosynihetic effects of mR162 in response to LNT stress in tomato. **(A)** Diagram of miR162 recombinant plasmid. *NPTIII* is the marker gene with kanamycin resistance on pRI101-AN vector. **(B)** Quanitative realime PCR analysis of miR162 in WT and transgenic lines. Data are mean and standard error of four independent experiments (n = 4), **P < 0.01 according to the t-test. **(C)** Morphological characterization of miR162 transgenic tomato plants (6-week whole plants, leaves, and flowers). **(D)** Maximum photochemical eficiency of photosystem II (Fv/Fm) in miR162 transgenic lines during the nine-day LNT stress treatment. **(E)** Stomatal parameters of mIR162 transgenic lines under LNT stress: net photosynthetic rate (*Pn*); intercellular Co2 concentration (*CI*); stomatal conductance (*Gs*): and transpiration rate (*Tr*). **(F)** Stomatal aperture and phenotype of miR162 transgenic tomato plants. Data are mean and standard error of four independent experiments (n = 4) with at least 50 individual stomata measured for each replicate. Scale bar = 5 mm. Control: 25°C/15°C, LNT stress: 25°C/15 (day/night temperature) Data are mean and standard error of four independent experiments (n = 4). Lowercase letters indicate significant differences at P< 0.05 according to Tukey's test.

To generate miR162-overexpressing and -silenced transgenic lines by using the tomato cultivar Ailsa Craig (AC), plasmids were extracted using a plasmid kit (TIANGEN, Beijing, China) and preserved in strains after extraction by the alkaline lysis method. Target fragments were recovered and purified with an agarose gel recovery kit (AXYGEN), and a carrier was recovered by double enzyme digestion. Top10 *Escherichia coli* competent cells were gently mixed with finishing products for culture. The miR162 precursor sequence has a typical hairpin structure, which can form an almost complete stem-loop structure. A recombinant plasmid was constructed by inserting miR162 into overexpressed and silenced pRI101-AN vectors, respectively. miR162 was inserted into pRI101-AN (SBS Genetech Co., Ltd, China), which plant expression binary vector by carrying a selectable marker gene NPT II (**Figure 1A**). Positive recombinant plasmids (pRI-miR162) were screened by PCR amplification using the corresponding primers (**Table S4**). The Sly-miR162 synthetic precursor was inserted into the prokaryotic expression pRI101-AN vector to construct the recombinant plasmid. The plasmid pRNAi-miR162 was transformed into LBA4404 by electroporation. Five miR162 overexpression and three miR162 silent single colonies were randomly selected for PCR and agarose gel electrophoresis. Primers for PCR identification were designed by kanamycin resistance marker gene NPTIII on pRI101-AN.

The recombinant plasmid was transformed into the *Agrobacterium tumefaciens* strain that was used to infect tomato leaves by electroporation. Eighty overexpression plants and 55 RNAi plants were generated by tissue culture, and these robust plants grew in the greenhouse.

Genomic DNA was extracted from tomato leaves of transformants using the Tissue DNA Kit (TIANGEN, Beijing, China) and was quantified based on spectrophotometric absorbance at 260/280 nm. For transgenic confirmation, isolated genomic DNA was used as templates to detect *NPT* II gene using PCR amplification (**Table S4**).

qRT-PCR analysis was performed to confirm silencing of miR162 using the SYBR Green Kit (TAKALA) in transgenic plants. RNA of tomato leaves was extracted by TRIzol. RNA reverse transcription was performed using the Reverse Transcription Kit (TAKALA).

DCL1-deficient mutants (*dcl1*) were generated with CRISPR/Cas9 technology using the tomato cultivar AC. The pYLCRISPR/Cas9 Pubi-H system manual was used to construct a CRISPR/Cas9-mediated knockout system (Ma et al., 2015). Briefly, Breaking-Cas web software was used to design the sgRNA target sequence (TACTCCACTACTACTTCTGA) within the coding region of the DCL1 (Solyc10g005130) gene. Because homozygous dcl1 is fatal (Castle et al., 1993), we obtained *dcl1-1-2/4/6/7/13/24* mutant tomatoes. Genomic DNA were extracted from tomato leaves to analyze DCL1 mutations with the Plant Genomic DNA Kit (TIANGEN, Beijing, China). DCL1 fragment was amplified using PCR, then sequencing results were compared to analyze the mutations. All primers used were listed in **Table S4**. The low temperature treatment method was the same as above.

### ABA extraction and determination

Extraction of ABA was performed with the protocol described by Xu et al. (Xu et al., 2018). Leaves were carefully weighed (0.5 g of fresh material) and ground into a fine powder with liquid nitrogen. Powdered samples were suspended in 10 mL extraction solvent (80% methanol with 1 mmol/L^−1^ butylated hydroxytoluene) at −20°C and incubated overnight at 4°C. Extracts obtained were vortexed and centrifuged for 10 min at 8,000 rpm. The pellets were extracted one more time with 5 mL extraction solvent and incubated for 1 h and centrifuged again under the same conditions. All supernatants were pooled and dried under a nitrogen stream. Extracts were treated with 5 mL petroleum ether for 15 min until decolorized. The resulting extracts were dissolved in 500 μL mobile phase (methanol with 3% glacial acetic acid).

ABA content was determined using high-performance liquid chromatography procedure (Waters e2695, USA). Water phase passed through a C18 column (250 mm × 4.6 mm, 5 μm particle size). It was eluted equivalently in methanol 3% glacial acetic acid aqueous solution (volume ratio 45:55). UV detection was acquired using wavelength of 265 nm, flow rate was 0.7 mL/min, column temperature was 25°C, and injection volume was 5 μL.

### Photosynthetic parameters and chlorophyll fluorescence

The photosynthetic parameters (*Pn:* photosynthetic rate, *Gs:* stomatal conductance, *Ci:* CO_2_ concentration, and *Tr:* transpiration rate) were measured by the GFS-3000 gas analyzer with the DUAL-PAM-100 measurement system. Light intensity photon flux density (PFD) was 1000 μmol·m^−2^·s^−1^ and the area of the standard measuring head used was 1.3 cm^2^ with atmospheric CO_2_ concentrations (approximately 500 ppm) and room temperature (Yang et al., 2020). Tomato plants were adapted in a dark environment for 30 min. Chlorophyll fluorescence of seedling was measured with the DUAL-PAM-100 measurement system (Heinz Walz, Effeltrich, Germany) at normal growth temperature. The intensity and duration of the light saturation pulse were 10,000 μmol photons·m^−2^·s^−1^ and 300 ms in measurement of the maximum photochemical efficiency of photosystem II (Fv/Fm).

### Stomatal aperture bioassays

Bioassays were performed on tomato plants at the six-week stage. The abaxial leaf epidermis was removed with tweezers and placed in Milli-Q water. Images of stomatal guard cells were captured using the Zeiss fluorescence inverted microscope (Axio Observer A1, Germany) and analyzed with the Zen 2012 software (Zeiss, Germany). A total of 200 stomata images of each treatment were composed of four biological replicates, and 50 stomata were randomly selected from each replicate for observation.

### Statistical analyses

ANOVA (analysis of variance) with SPSS software v17.0 (SAS Institute, Cary, NC, USA) were used to do statistical analyzes, and the mean values were presented with standard deviation of four biological replicates, which compared by the to the one-way ANOVA comparison test at the probability level of *P* < 0.05 or 0.01. All graphs were created using Prism 8 software (GraphPad, CA, USA).

## Results

### sRNA profiles and identification of ABA-associated miRNAs under LNT stress

**Figure 2:**
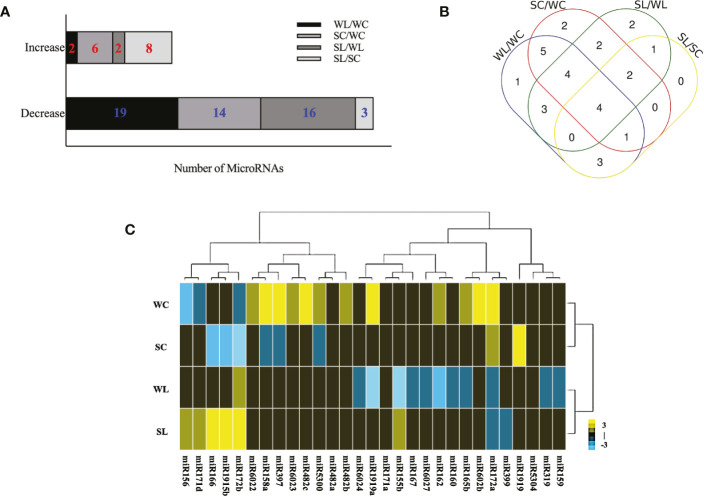
SRNA-seq profiing of differentially expressed mRNAS under LNT stress in tomato. **(A)** Diferential expression of conserved miRNAs in four libraries (WC, wild-type tomato plants under normal conditions; WL, wild-ype tomato plants under LNT stress; SC, *sit* tomato mutans under normal condilons; SL, *sit* tomato mutans under LNT stress). Data are means and standard error of four independent experiments (n = 4). Significant differences compared with each group was determined using *P* < 0.05. **(B)** Venn diagram of significantly different miRNAS in the four groups. **(C)** Clustering of diflferentially expressed miRNAs. Yellow indicates high expression, while blue indicates low expression. The consecutive fold change was set from -3 to 3.

To identify which miRNAs responded to LNT stress and were involved in the ABA-dependent signaling pathway in tomato, four independent sRNA libraries were constructed for deep sequencing, including samples of WT tomato plants under normal conditions (WC), WT tomato plants under LNT stress (WL), *sit* tomato mutants under normal conditions (SC), and *sit* tomato mutants under LNT stress (SL). The sRNA libraries yielded 32,338,934 raw reads on average (**Table S1**). miRNAs mainly gathered in 23-nt and 24-nt in response to LNT stress (**Figure S1**). After removing low-quality raw reads, contaminated adapter sequences, and poly A tail sequences, the related miRNAs were identified with 19,290,244 clean reads. These miRNA sequences were compared with the miRNA precursor and mature sequences that were known in miRbase 22.1 (**Table S2**). Except for known miRNAs, 299 new miRNAs were predicted with criteria that included the presence of the hairpin structure of miRNA precursors, reverse sequence of initial transcription of miRNAs, and Dicer enzyme of mature miRNAs. Secondary structures of new miRNAs were predicted by RNAFold (**Figure S2**). Among conserved miRNAs that were differentially expressed genes (DEGs) in the comparison of the four groups, twenty-one miRNAs were regulated by LNT stress (WL/WC), in which nineteen miRNAs were decreased and two miRNAs were increased. Among twenty DEGs, fourteen decreasing miRNAs and six increasing miRNAs were individually modulated by ABA under normal conditions (SC/WC). Eighteen DEGs were changed in the comparison of WT and *sit* mutants with sixteen increasing miRNAs and two decreasing miRNAs (SL/WL). Eleven DEGs were significantly changed in response to LNT stress and depended on the ABA signaling pathway, in which three miRNAs were decreased and eight miRNAs were increased (SL/SC) (**Figure 2A**). Venn diagram analysis demonstrated that four known miRNAs were ubiquitous in all groups, including *miR160a, miR162, miR1919b*, and *miR5300* (**Figure 2B**). The pattern cluster of twenty-nine DEGs showed which miRNAs have similar regulation and biological characteristics in each cluster. Upregulated miRNAs are represented with yellow squares, and downregulated miRNAs are represented with blue squares (**Figure 2C**). Gene ontology (GO) enrichment analysis of significantly changed miRNAs suggested that the LNT-related miRNAs were mostly associated with the cell and membrane cellular components. Furthermore, they participated in 16 biological processes in LNT resistance in tomato (**Figure S3**).

**Figure 3:**
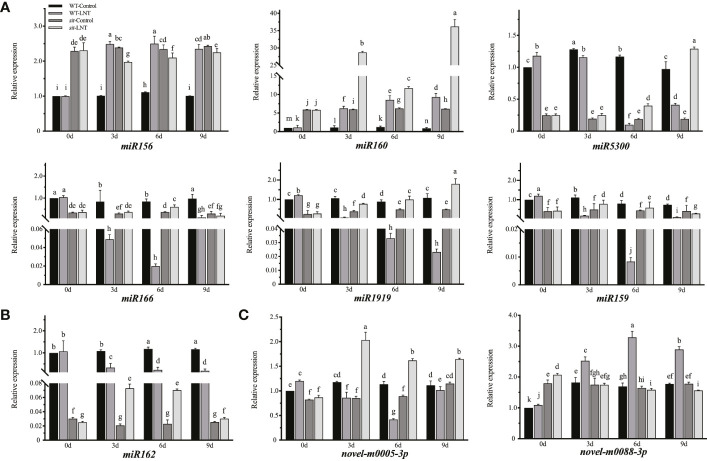
Mature miRNA changes in response to LNT stress with the ABA-dspendsnt signaling pathway in tomato. **(A)** Relative expression of known miRNAS (*miR156, miR160, miR5300, mIR166, mIR1919,* and *miR159*) in succession of LNT treatment. **(B)** Relative expression of *miR162* as the candidate miRNA. **(C)** Relative expression of new mIRNAS (*novel-m0005-3p* and *novel-m0082-3p*) in succession of LNT treatment. d, Days of LNT treatment (0, 3, 6, and 9 days). Control: 25 °C/15 °C, LNT stress: 25 °C/6 °C (daylight temperature). Data are means and standard error of four independent experiments (n = 4). Lowercase letters indicate significant differences at *P* < 0.05 according to Tukey's test.

Among differentially expressed miRNAs, seven known miRNAs (*miR156, miR160, miR5300, miR166, miR1919, miR159*, and *miR162*) and two novel miRNAs (*novel-m0005-3p* and *novel-m0088-3p*) were quantified by real-time RT-PCR on the basis of the sRNA-seq results (**Figure 3**). *miR159* expression in *sit* mutants was significantly changed under LNT stress in the real-time RT-PCR results, but not in the sRNA-seq results. *miR162* was significantly decreased in WL than that in WC after LNT stress, which suggested that miR162 was a negative regulator in response to LNT stress. However, miR162 expression increased under LNT stress in *sit* mutants, indicating that the function of miR162 was disturbed without ABA biosynthesis in LNT response (**Figure 3B**). Specifically, miR162 participated in LNT resistance negatively with the ABA-dependent signaling pathway in tomato. Notably, miR162 expression was downregulated in *sit* mutants under normal conditions (**Figure 3B**). Compared with miR162, the fold change of the other three miRNAs (miR160, miR1919 and miR5300) that also respond to LNT stress and ABA (**Figure 2B**) were significantly less than 51.21 times of miR162, and the miR162 expression trend of LNT response was opposite to that of WT in *sit*. It is interesting that ABA might regulated the miR162 mechanism in response to LNT stress in tomato.

### miR162 regulates stomatal conductance in response to LNT stress

**Figure 4:**
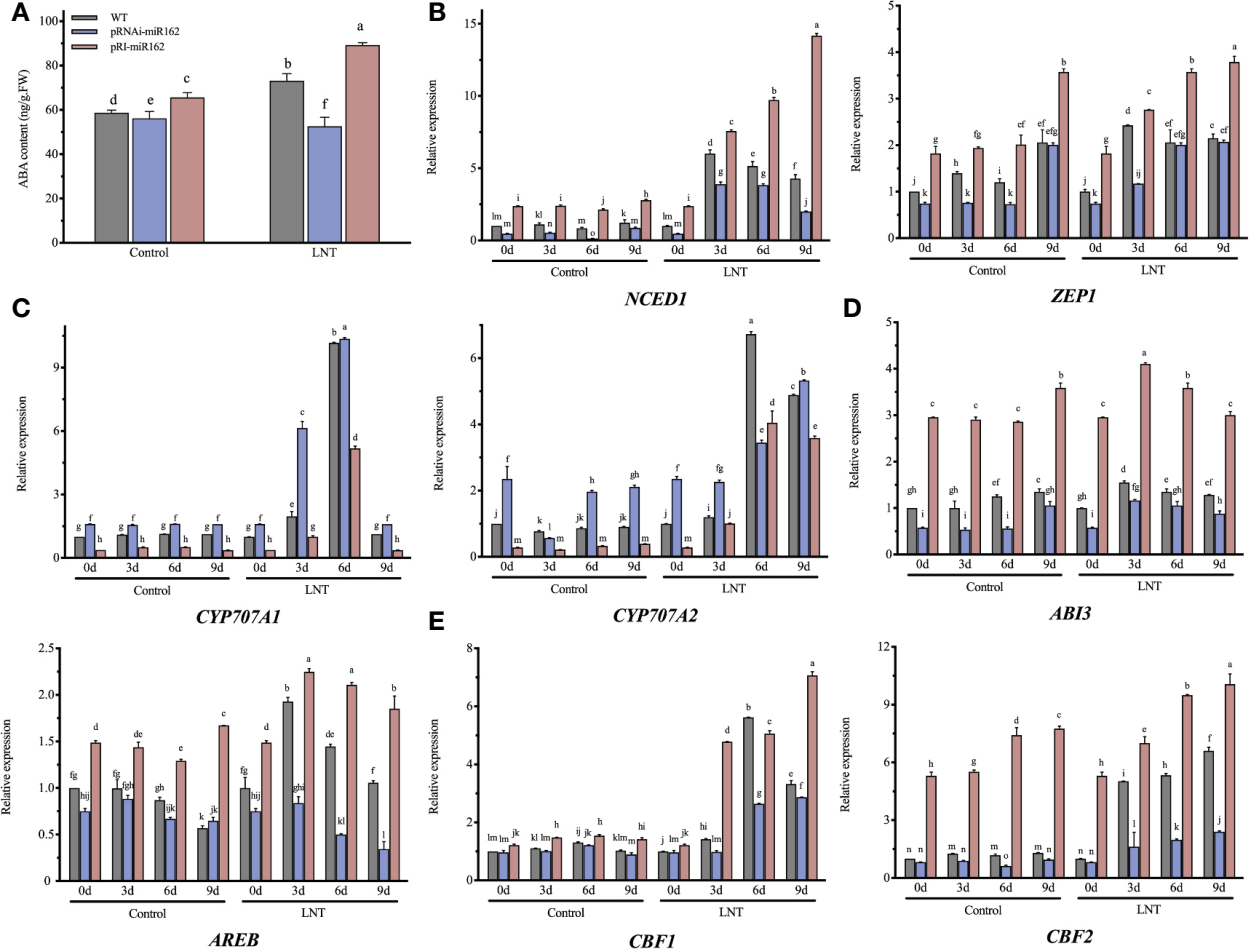
Effects of mIR162 on the ABA signaling pathway in response to LNT stress. **(A)** ABA content of miR162 transgenic lines under LNT stress. **(B)** Expression of ABA biosynthesis genes in miR162 transgenic lines during LNT stress. **(C)** Expression of ABA catabolism genes in mIR162 transgenic lines during LNT stress. **(D)** Expression of ABA responsive genes in miR162 transgenic lines during LNT stress. **(E)** Expression of ABA-related cold-induced genes in miR162 transgenic lines during LNT stress. **(D)** Days of LNT treatment (0, 3, 6, and 9 days). Control: 25°C/15°C, LNT stress: 25°C/6°C (day/night temperature). Data are mean standard error of four independent experiments (n = 4). Lowercase letters indicate significant ferences at *P*< 0.05 according to Tukey's test.

To examine the negative functions of miR162 in response to LNT stress, we obtained 55 RNAi and 80 overexpression transgenic tomato plants by ligating the *sly-miR162* precursor into the pRI101-AN vector (**Figure 1A**). According to the marker gene *NPTIII*, conferring kanamycin resistance, transgenic adult plants were confirmed by DNA and RNA analysis. Three RNAi and five overexpression tomato transgenic lines were identified. Compared with WT, miR162 expression in RNAi tomato plants (pRNAi-1-1, pRNAi-2-2, and pRNAi-2-59) was significantly downregulated by 5.1, 4.7, and 1.8-fold, respectively. miR162 expression in pRI-1-5, pRI-1-6, pRI-1-7, pRI-2-8, and pRI-2-10 was significantly higher than that in WT, upregulated by 10, 13.7, 12.4, 15.1, and 7-fold (**Figure 1B**). Morphological analysis of miR162 transgenic plants showed that plant growth and leaf morphology were affected by miR162 expression (**Figure 1C**). pRNAi-miR162 plants were stronger with thicker stem compared with the easily breakable tall and slender stem in WT plants. pRNAi-miR162 plants had lesser leaf margin serrations than WT plants. However, the changes in flower morphology and florescence were not significant between pRNAi-miR162 and WT plants. The plant growth and flower morphology in pRI-miR162 plants did not change significantly compared with WT plants. Green leaves are the main organs of plant photosynthesis. As changes of miR162 transgenic plants, we sought to elucidate the role of miR162 in the photosynthesis of tomato. The statistical analyzes is based on average data from three pRNAi and pRI transgenic lines in **Figures 1**, **4**. The maximum photochemical efficiency of photosystem II (Fv/Fm) was elevated in pRNAi-miR162 under LNT stress, but it did not significantly change in pRI-miR162 compared with WT (**Figure 1D**). For nine days of LNT treatment, the most significant difference was observed on the third day among miR162 transgenic lines, which was further analyzed for photosynthetic performance. Under LNT stress, Fv/Fm was significantly reduced in pRI-miR162, even lower than that in WT. However, Fv/Fm was no significant change in comparison of control and LNT stress in pRNAi-miR162. The similar trends with Fv/Fm were shown in gas exchange parameters of tomato that pRNAi-miR162 had higher *Pn* than WT under LNT stress (**Figure 1E**). Under LNT stress, both intercellular *Ci* and *Tr* increased in pRNAi-miR162 but were significantly decreased in pRI-miR162 compared with WT. The same trends between *Pn* and *Ci* indicated that stomatal restriction limited photosynthetic rate of miR162 transgenic lines in response to LNT stress. Specifically, stomata opened more in pRNAi-miR162 than in WT under LNT stress (**Figure 1F**), indicating a high *Gs* in pRNAi-miR162 (**Figure 1E**). Therefore, miR162 negatively modulated LNT tolerance in tomato by regulating stomatal conductance.

### miR162 participated in the ABA signaling pathway to mediate LNT responses

Based on the four sRNA-seq libraries, ABA inhibited miR162 to regulate the LNT response in tomato. The feedback relationship was hypothesized between miR162 and ABA. To elucidate how miR162 regulates the ABA signaling pathway in response to LNT stress, the ABA content was elevated in miR162 transgenic lines (**Figure 4A**). Under LNT stress, ABA content was increased to mediate LNT resistance of tomato. However, it was inhibited in pRNAi-miR162, but was enhanced in pRI-miR162 compared with WT. miR162 might positively regulate ABA as feedback in response to LNT stress. The expression of ABA biosynthesis genes (*NCED1* and *ZEP1*) decreased in pRNAi-miR162 but increased in pRI-miR162 during LNT stress (**Figure 4B**). In contrast, both ABA catabolism genes (*CYP707A1* and *CYP707A2*) had significantly lower expression in pRI-miR162 but had higher expression in pRI-miR162 than in WT (**Figure 4C**). The increase in ABA biosynthesis and decrease in ABA catabolism resulted in ABA accumulation in pRI-miR162, indicating that miR162 regulated the ABA content of tomato plants and participated in the ABA signaling pathway in response to LNT stress. As regulators involved in the ABA response mechanism, the expression levels of *ABI3* and *AREB* were downregulated like the ABA content of pRNAi-miR162 but were upregulated in pRI-miR162 under LNT stress (**Figure 4D**). The feedback regulation of miR162 and the ABA signaling pathway might regulate ABA-depended LNT resistance to preserve plant defense in homeostasis of tomato. Indeed, further evidence has showed that miR162 regulated LNT resistance by the ABA signaling pathway. Both CBF1 and CBF2 can respond to LNT and ABA (Knight et al., 2004), and the expression levels of *CBF1/2* (examined by qRT-PCR) were higher in pRNAi-miR162 compared with WT and pRI-miR162 (**Figure 4E**). These results demonstrate that miR162 played a negative role in the ABA signaling pathway in response to LNT stress and provided feedback regulation of ABA content, maintaining homeostasis in response to diverse abiotic stresses.

### miR162 modulates DCL1 cleavage in response to LNT stress at the post-transcriptional level

**Figure 5:**
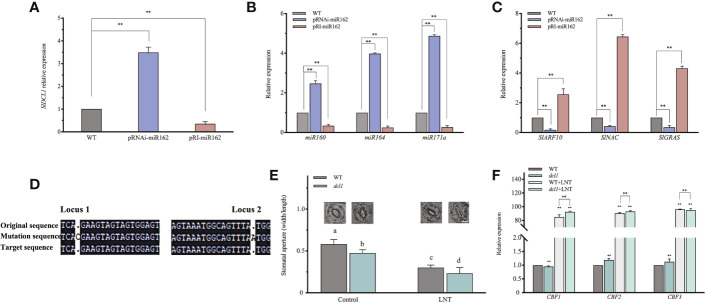
mIR162 regulated DCL1 cleavage and participated in the ABA signaling pathway in response to LNT stress. **(A)** DCL 1 expression in miR162 transgenic lines. **(B)** Expression analysis of DCL1-cleavable miRNAs (*miR160, miR164*, and *miR171a*) in miR162 transgenic lines. **(C)** Expression analysis of target genes (*ARF10, NAC*, and *GRAS*) of DCL1-cleavable miRNAS in miR162 transgenic lines. **(D)** Monoclonal sequencing of SIDCL1 mutant identification (*dcl1-1-4/6/7*). **(E)** Stomatal aperture and phenotype of *dcl* tomato plants under LNT stress. Data are mean and standard error of four independent experiments (n = 4) with at least 50 individual stomata measured for each replicate. Scale bar = 5 μm. **(F)** Expression analysis of ABA-dependent cold tolerance genes (*CBF1, CBF2*, and *GBF3*) in *dcl* tomato plants under LNT stress. Control: 25°C/15°C, LNT stress: 25°C/G°C (dayinight temperature). Data are mean and standard error of four independent experiments (n = 4). Lowercase letters indicat significant differences at P < 0.05 according fo Tukey's test. **P< 0.01 according o the test.

miRNAs mediated mRNA degradation of target genes and inhibited targeted mRNA translation to regulate gene expression in response to environmental stress (Xie et al., 2015). The miR162 target sequence might be near the middle of DCL1 mRNA, which was subjected to negative feedback regulation by miR162 activity (Xie et al., 2003). To investigate whether miR162 regulated DCL1 in LNT resistance of tomato, SlDCL1 expression was evaluated by real-time PCR. It was upregulated in pRNAi-miR162 and downregulated in pRI-miR162, indicating negative regulation between miR162 and DCL1 (**Figure 5A**). DCL1, as the major producer of miRNAs, is involved in post-transcriptional regulation in response to a series of stresses (Park et al., 2002). We examined whether DCL1 cleavage was regulated by miR162. DCL1-cleavable miRNAs (miR160, miR164, and miR171a) (Kravchik et al., 2014b) were highly expressed in pRNAi-miR162 compared with WT (**Figure 5B**), and their corresponding target genes (ARF10, NAC, and GRAS) had opposite expression levels in pRNAi-miR162 (**Figure 5C**; **Table S3**). miR162 negatively modulated DCL1 cleavage, which inhibited the responses of SlARF10, SlNAC, and SlGRAS by increasing miR160, miR164, and miR171a at the post-transcriptional level.

**Figure 6:**
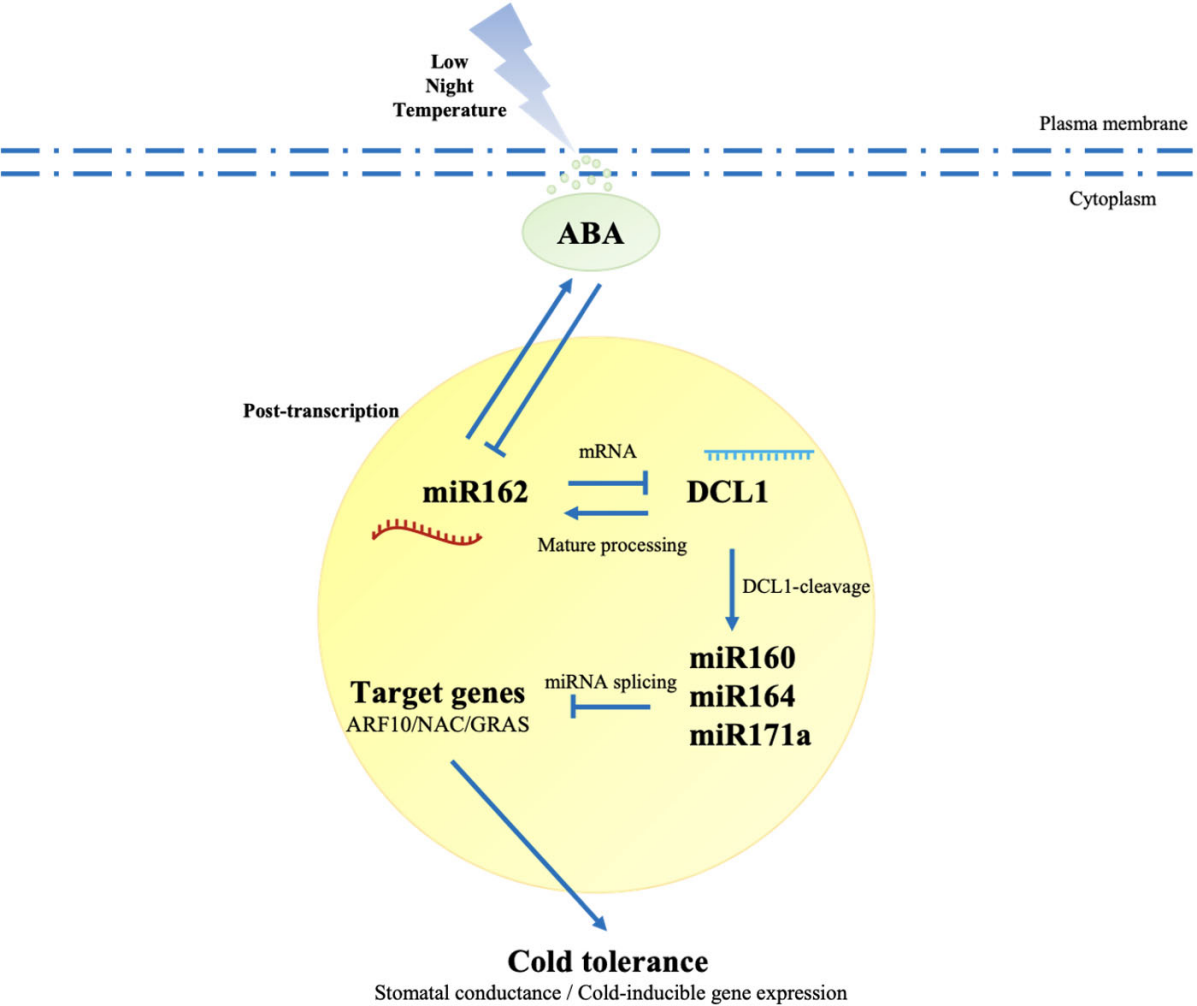
miR162-mediated DCL1 cleavage regulated cold tolerance with the ABA-dependent signaling patfway in tomato. Under LNT stress, ABA was activated to mediate multiple responses of tomato plants, including the negative regulation of miR162. Post-transcription was a crucial process in response to environmental stress. miR162 inhibited DCL1 cleavage by targeting the mRNA of DCL1. In DCL1 cleavage, DCL1 mediated miRNAs (miR160, mIR164, and miR171a) and their targets (ARF10, NAC, and GRAS) to regulate the expression of cold-inducible genes and stomatal movement under LNT stress. DCL1 is in the processing of mature miRNAs, including mIR162, which provides feedback to the ABA signaling pathway, so tomato plants resist LNT stress and maintain equlibrium. Sold lines represent regulatory links overserved in the network. Arrows indicate positive regulation, and blunt-ended bars indicate inhibition. A line does not necessarily represent unique or direct regulation by miR162 In the post-transcriptional process in cold stress response.

Previous studies have suggested that miRNAs are strategically located in negative, positive, and double-negative feedback loops to mediate stress responses (Leung and Sharp, 2010). DCL1-deficient mutants (dcl1) were generated to examine the adaptive mechanisms of miR162-DCL1 post-transcription loops in LNT resistance. Homozygous mutation of DCL1 leads to embryo death in Arabidopsis (Castle et al., 1993), and the same embryo death phenomenon might exist in tomato because of a mutation in SlDCL1. The mutation of SlDCL1 was obtained by CRISPR/cas9. In dcl1 plants, the gene of a sister chromosome of SlDCL1 was edited (single base deletion and addition), which affected the mRNA encoding the protein. Monoclonal sequencing indicated that dcl1-1-4/6/7 belonged to the same tomato strain. In the coding region, loci 1 and 2 had another base addition that led to the partial or total loss of function of SlDCL1 (**Figure 5D**). Under LNT stress, stomatal apertures of dcl1 plants were significantly reduced compared with WT plants, which had a phenotype similar to pRI-miR162 (**Figure 5E**). Similarly, in the response mechanism of miR162, SlDCL1 was involved in the ABA signaling pathway and regulated ABA-dependent cold tolerance genes (CBF1, CBF2, and CBF3) that were upregulated in dcl1 plants under LNT stress (**Figure 5F**). The ABA signaling pathway and cold resistance of tomato might have been activated with DCL1 cleavage. Taken together, the post-transcriptional mechanism of miR162 in response to LNT stress was determined. With DCL1 regulating the processing of mature miR162, miR162 conversely inhibited DCL1 cleavage, which modulated miRNAs and miRNA targets to regulate resistant gene expression and cold tolerance by the ABA-dependent signaling pathway under LNT stress. Besides, the feedback regulation between the post-transcriptional process of miR162 and ABA maintained resistance to LNT stress in tomato plants (**Figure 6**).

## Discussion

### miR162 plays a negative role in response to LNT stress

miRNAs are non-coding RNAs (ncRNAs) that respond to abiotic stress in plants. They act on target genes to regulate plant growth and development in stress resistance (Zhang, 2015). Cold stress not only affects the geographical distribution and planting season of crops but also destroys their normal activities causing plant damage (Chinnusamy et al., 2007). miRNAs are involved in cold resistance in plants and play key roles in response to cold stress (Zhang et al., 2009). Following high-throughput sequencing, 192 increased and 205 decreased miRNAs are identified in response to chilling stress in leaves of *S. habrochaites* seedlings (Cao et al., 2014). It is worth comparing that the role of miR167, miR169, miR172 and miR393 has been confirmed in tomato cold resistance, which are activated in the early time points of cold treatment (Koc et al., 2015). We verified 21 known miRNAs in response to LNT stress, a common stress of tomato during winter. Using miRNA microarrays and deep sequencing technologies (<xr rid="r70">Zhang et al., 2014</xr>), most miRNAs decreased under LNT stress (**Figure 2A**). The responsive miR162 inhibited cold tolerance *via* the ABA signaling pathway in tomato (**Figure 3B**). This result was consistent with relevant studies of miRNA in rice, in which 24 miRNAs responded to cold stress, including miR1425, which played a negative role in the improvement of cold tolerance in rice. In contrast, *miR393* expression continuously increased to cope with cold stress (Jeong and Green, 2012). Indeed, different miRNAs play different roles in response to cold stress. The expression of the *miR319* family positively correlated with cold tolerance in tomato (Chen et al., 2015b). Compared with WT tomato, *ShamiR319d* overexpression significantly enhanced stress resistance with weaker photoinhibition of PS II and higher scavenging activity of reactive oxygen species (Shi et al., 2019a). miR319 regulated TCP3/TCP29, which is involved in Ca^2+^ transduction and anthocyanin biosynthesis in response to cold stress. MBSI, a MYB binding site, was found in the promoter regions of miR319a and miR319b, indicating that miR319 targeted MYB65 and was involved in flavonoid biosynthesis regulation and mediated cold tolerance (Shi et al., 2019b). It is concluded that sly-miR166 acts as a cold-inducible switch that regulates SlHB15A levels in the ovule (Clepet et al., 2021). In our study, the photosynthetic efficiency was elevated by the stomatal opening with *SlmiR162* deficiency (pRNAi-miR162) that targeted DCL1 cleavage and DCL1-cleavable miRNAs (miR160, miR164, and miR171a) in response to LNT stress (**Figures 1D, E**; **5A–C**). Besides cold stress, miRNAs respond to various stress treatments; for example, miR472b and miR530 play positive roles in poplar under hypoxia stress (Ren et al., 2012). *miR168*, *miR408*, and *miR396* expression decreased during drought (Zhou et al., 2010), but miR156 expression was induced by salt stress in Arabidopsis (Liu et al., 2008). The reason might be the different growth environments of different plants (Zhang et al., 2014). Even in the same plant species, differential gene expression might be due to individual selection of their WT relatives under same stress. Although several plant miRNAs have been identified by sequencing, their functions in stress response are little known. To investigate the biological functions of miRNAs and predict their corresponding target genes by proteome sequencing can provide a molecular basis for environmental stress resistance in crop breeding.

### miR162 mediates DCL1 cleavage to regulate gene expression at the transcriptional and post-transcriptional levels

miRNA regulates gene expression by three mechanisms-RNA cleavage, translation inhibition, and transcriptional silencing. Most plant miRNAs are highly complementary to target mRNAs that encode TFs or other functional proteins. miRNAs are the main factors and hold an important position in the core of the regulatory network. For example, leaf development was affected by *miR319b* deficiency but recovered after *TCP3* inserted, indicating that miR319b regulates plant biological functions by targeting *TCP3* mRNA in Arabidopsis (Koyama et al., 2017). The encoded transcripts of *SlIPT2* and *SlIPT4* undergo sly-miR208-guided cleavage in the leaves of *35S::pre-miR208* plants, which revealed their targeting by this putative miRNA (Zhang et al., 2020). In addition, miRNA targets F-box proteins and ubiquitin-binding enzymes, which are related to proteasome degradation, to regulate protein stability. DCL1 is necessary for miRNA accumulation as an RNase endonuclease, which splices pri-miRNA to form mature miRNA. DCL1 mRNA is also subject to negative feedback regulation by miR162 activity (Xie et al., 2003). To characterize the negative relationship between miR162 and DCL1 in tomato, *SlDCL1* expression was evaluated in miR162 transgenic lines (**Figure 5A**). This feedback mechanism suggests that miRNA regulated its biogenesis and function in plants. Similarly, miR168 targets *AGO1* mRNA (Vaucheret et al., 2004) and miR403 targets *AGO2* mRNA (Allen et al., 2005), and these proteins contain a PAZ sRNA binding domain and a PIWI–RNase H-like domain, respectively. Proteins such as DCL1, AGO1, HEN1, and HYL are fundamental to miRNA effector pathways identified during genetic screening for developmental defects in pleiotropic phenotypes (Hunter et al., 2003). For example, the most severe mutations in the DCL1 mutant led to early embryonic arrest and pleiotropic defects, including abnormalities in flower organogenesis, leaf morphology, and axillary meristem initiation (Schauer et al., 2002). In the present study, a homozygous mutation in *SlDCL1* led to embryo death in tomato. Although these developmental defects might be caused by impaired miRNAs, the disruption of the other pathways also reflects the action of these functional genes in the generation and function of sRNA. However, the specific regulatory effect still needs further research in plants.

miRNAs do not function directly in response to stress but are indirectly involved in the complex gene networks and regulate key components (Winter and Diederichs, 2011). miR162 mediated DCL1 cleavage negatively by regulating DCL1-cleavable miRNAs (miR160, miR164, and miR171a) and their corresponding target genes (ARF10, NAC, and GRAS) at the post-transcriptional level (**Figures 5B, C**). Recent studies have suggested that the post-transcriptional modification of NAC domain TFs play a positive role in abiotic stress tolerance (Redillas et al., 2012). In Arabidopsis, miR156 targeted *SPL*, which increase and preserve defense against heat stress that recurs later (Stief et al., 2014). Because of high complementarity between miRNA and target mRNA (Kravchik et al., 2014a), miRNA-mediated cleavage has been the main action of miRNA regulation. Although miRNA-mediated cleavage has been applicable to many of these targets *in vivo*, both regulatory mechanisms of miRNAs responded to complex environmental stress at the transcriptional and post-transcriptional levels, and the biological significance of target genes regulated by miRNA still need to be explored.

### miR162 participates in the ABA signaling pathway to activate LNT resistance in tomato

The initial increase in ABA is a crucial regulatory mechanism when plants experience cold stress (Knight et al., 2004). Previous studies have revealed that miRNAs can respond to ABA treatment (Sunkar and Zhu, 2004). Based on reverse genetics, miR162 was identified as a key response factor that played negative roles in the ABA-dependent resistance pathway under LNT stress (**Figure 3B**). miR162 transgenic lines were obtained to evaluate how miR162 mediates LNT response in combination with the ABA signaling pathway. miR162 deficiency inhibited biosynthesis and enhanced catabolism of ABA as a feedback regulation to maintain homeostasis under LNT stress (**Figure 4**). Correspondingly, the expression of ABA biosynthesis genes (*OsNCED1*/*OsNCED3*) were increased in miR164b-resistant OsNAC2 overexpression lines of rice (Redillas et al., 2012). The same conclusion in maize showed that miR168 and miR528 are inhibited by MAPK and peroxidase genes inducted in the ABA signaling pathway (Wei et al., 2009). Similarly, the expression of drought-suppressed *miR169* was inhibited by an ABA-dependent pathway (Li et al., 2008). Indeed, miRNAs participate in stress response by regulating their downstream targets in ABA response, auxin signaling pathway, osmotic protection, and antioxidant system (Xu et al., 2014). Consistent with a study in *A. thaliana*, miR167a targets PpAPF8 and is involved in the auxin signaling pathway and regulates fruit development in peach (Kasschau et al., 2003). ARF10 can induce ABA accumulation by promoting ABA synthesis ABI5 gene expression in tomato (Liu et al., 2016). SlmiR160 might target SlARF10 to regulate stomatal closure, which reduces water loss and maintains water balance of leaves through the ABA signaling pathway. The association of miRNAs and ABA was demonstrated in tomato before; miR156 is an ABA-dependent regulator on the after-effect of drought on stomata (Visentin et al., 2020). Studies in the past decade have associated miRNAs with the development of guard cells and the regulation of stomatal movement, especially in stress conditions (Ding et al., 2013; Curaba et al., 2014). Under LNT stress, miR162 regulated stomatal closing to affect photosynthesis in tomato (**Figures 3D–F**). Similarly, miR169c reduces stomatal opening, thereby enhancing drought tolerance in tomato (Zhang et al., 2011). At the post-transcriptional level, miRNA promotes stomatal development by targeting AGO1 (Jover-Gil et al., 2012). *miR171c* overexpression was sensitive to ABA treatment and increased stomatal density, indicating that miR171c regulates stress gene expression and stomatal development by the ABA pathway (Yang et al., 2017). Taken together, these results suggest that miR162 regulated stomatal conductance and modulated ABA accumulation in LNT-responsive networks. Except for TFs and response behaviors, stress response genes are regulated by miRNAs in plants. The C-repeat binding factor (CBF) family not only responds to cold stress (Gilmour et al., 1998) but is also involved in the ABA signaling pathway (Knight et al., 2004). As we presented, *CBF1/CBF2* expression decreased in pRNAi-miR162, enabling the improvement of cold tolerance in tomato (**Figure 4E**). Indeed, ABA-dependent cold tolerance genes (*CBF1, CBF2*, and *CBF3*) were upregulated in *dcl1* plants (**Figure 5F**). The ABA signaling pathway and cold resistance of tomato might have been activated with DCL1 cleavage under LNT stress. On analysis of the *cis*-elements of the DCL promoter, all DCL family members had hormone response elements. For example, AtDCL1 silencing leads to the hypersensitivity of seeds to ABA during Arabidopsis seed germination. The expression of ABA-related and stress-induced genes increased in *dcl2* mutant and *dcl2/3/4* triple mutants, including ABF3, DREB1A, KIN2, and RAB18 (Zhang et al., 2008). Further analysis of miRNA biogenesis genes are indicated that not only ABA and salt sensitivity but also the expression level of ABA response factors (ABI3, ABI4, and ABI5) are enhanced in mutant plants (Song et al., 2013). It is not clear whether ABA-related TFs affect the formation of DCL and miRNA. The relationship between phytohormones and the synergistic regulation of miRNA biogenesis can be a research direction in the future.

Phytohormones are at the heart of regulatory networks that respond to environmental stressors (Peleg and Blumwald, 2011). Here, we proposed a model of post-transcription and ABA (**Figure 6**). These rapid processes are based on initial ABA response to cold stress and maintains the ABA signaling pathway to improve cold tolerance. Most miRNAs and their targeted TFs are highly evolutionarily conserved in plants (Axtell and Bartel, 2005). Therefore, plants may use regulatory mechanisms at the post-transcriptional level to quickly respond to stress conditions. miRNA-mediated gene targeting combined with phytohormones might be a mechanism common to all plant species in response to environmental stresses.

## Conclusion

SlmiR162 was identified from known miRNAs that have differential expression in four sRNA libraries connected with LNT stress and the ABA signaling pathway. miR162 was involved in the LNT responsive pathway by indirectly regulating stomatal conductance and photosynthesis. A clear negative correlation was observed between miR162 and its target DCL1. Post-transcriptional interaction with the ABA signaling pathway occurred in the regulation of cold resistance. Confirmed with DCL1-deficient mutants, miRNA160/miRNA164/miRNA171a were reduced by miR162 with DCL1 cleavage. miR162 acted as a negative regulator and participated in the ABA signaling pathway of cold tolerance in tomato. The model of the association between ABA and post-transcriptional processes could be used to understand the complex mechanism of plant resistance that eventually impacts crop yield under abiotic stress.

## Data availability statement

The data presented in the study are deposited in the Zenodo repository, accession number DOI: 10.5281/zenodo.7659370.

## Author contributions

YYL performed the experiments, analyzed the data, and participated in the drafting. YL and ZG carried out the experiments. FW, XT, and MQ coordinated the study. YFL and TL designed the experiments, granted funds, and revised the manuscript. All authors contributed to the article and approved the submitted version.

## Supplementary material

Supplementary Figure 1 Small RNA library sequencing fragment length.

Supplementary Figure 2 Prediction and secondary structure of new miRNAs.

Supplementary Figure 3 GO categories of differentially expressed miRNAs in the four groups.

Supplementary Table 1 Overview of the high-throughput sequenced sRNA libraries.

Supplementary Table 2 Comparison of miRbase.

Supplementary Table 3 Promoter cis-acting elements analysis of DCL1-cleaved miRNAs target genes.

Supplementary Table 4 All primers used for quantitative RT-PCR.
