# Supplementary figures and images for: MicroRNA162 regulates stomatal conductance in response to low night temperature stress via abscisic acid signaling pathway in tomato
Source: Front Plant Sci. 2023 Mar 2;14:1045112. doi: 10.3389/fpls.2023.1045112 (PMC10019595; doi:10.3389/fpls.2023.1045112)

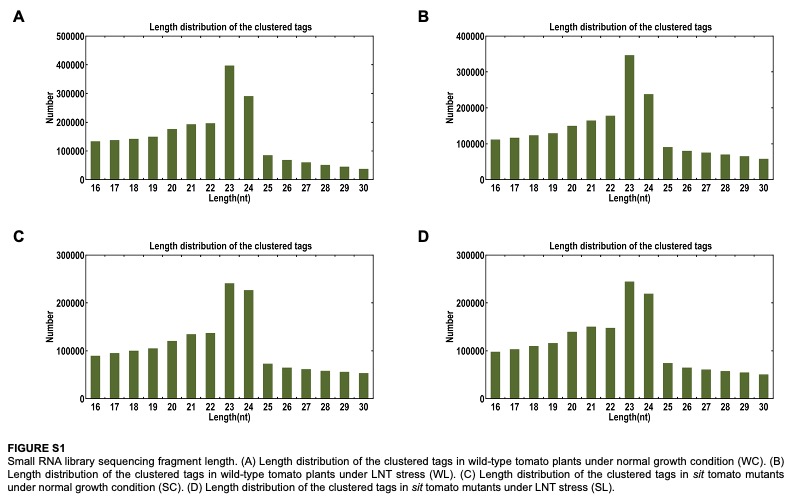

Supplement: Supplementary Figure 1 — Small RNA library sequencing fragment length. [file Image_1.jpeg]

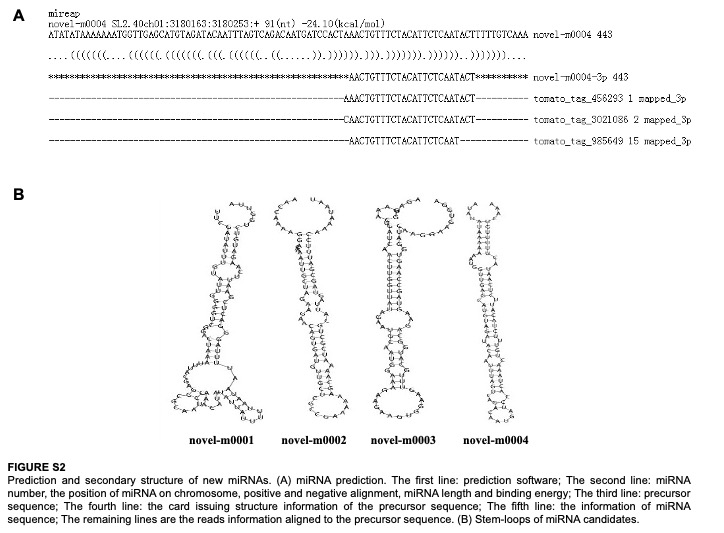

Supplement: Supplementary Figure 2 — Prediction and secondary structure of new miRNAs. [file Image_2.jpeg]

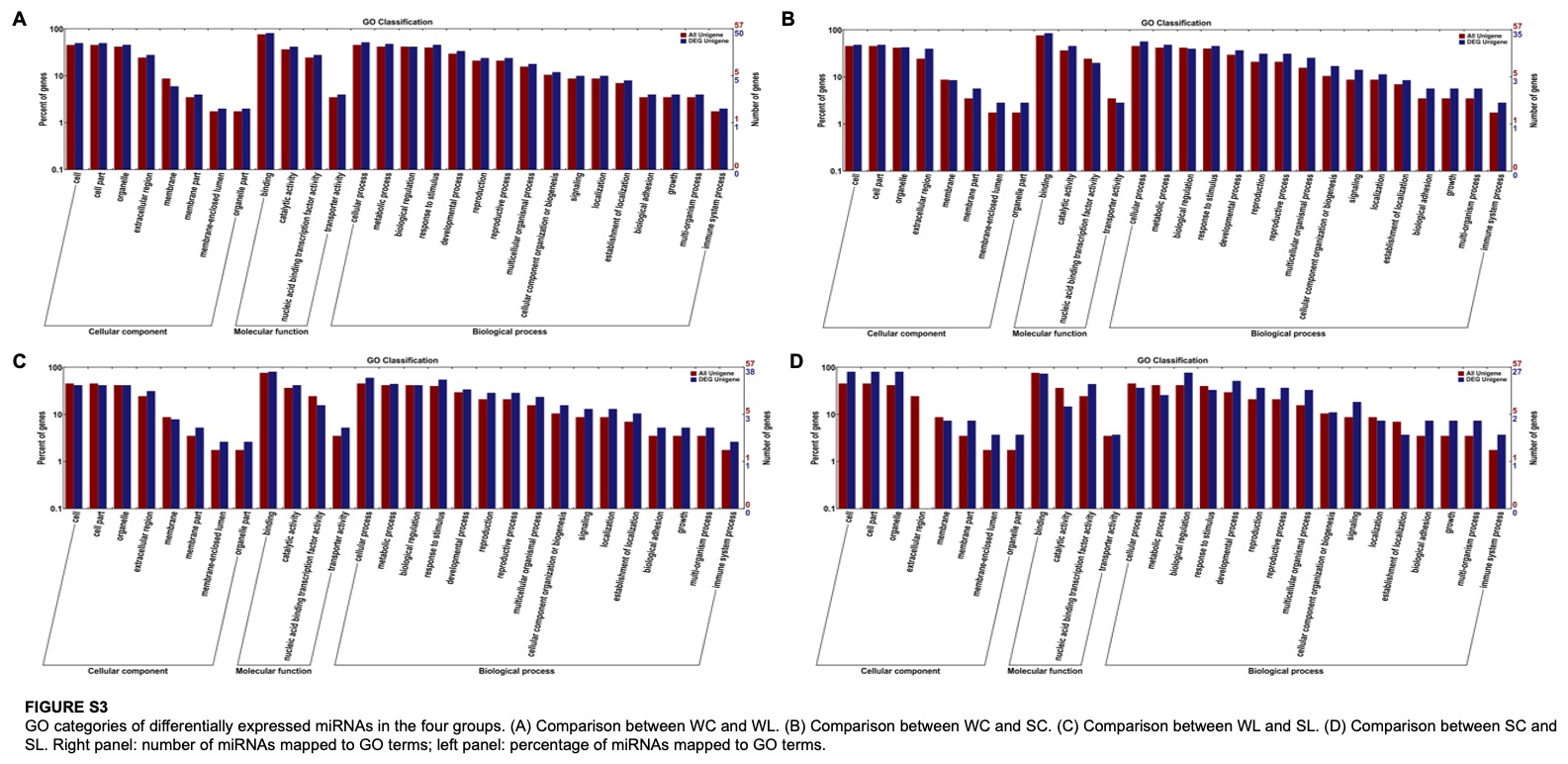

Supplement: Supplementary Figure 3 — GO categories of differentially expressed miRNAs in the four groups. [file Image_3.jpeg]
